# *Escherichia coli* YmdB regulates biofilm formation independently of its role as an RNase III modulator

**DOI:** 10.1186/1471-2180-13-266

**Published:** 2013-11-24

**Authors:** Taeyeon Kim, Juyeon Lee, Kwang-sun Kim

**Affiliations:** 1 Superbacteria Research Center, Korea Research Institute of Bioscience and Biotechnology (KRIBB), 125 Gwahak-ro, Yuseong-gu, Daejeon 305-806, Korea; 2 Department of Molecular Biology, Chonbuk National University, 567 Baekje-daero, Deokjin-gu, Jeonju-si, Jeollabuk-do 561-756, Korea; 3 Department of Biosystems and Bioengineering, University of Science and Technology, 217 Gajung-ro, Yuseong-gu, Daejeon 305-350, Korea

**Keywords:** Ribonuclease III (RNase III), Microarray, Biofilm, Trans-acting regulator, *Escherichia coli*

## Abstract

**Background:**

Ribonuclease III (RNase III) activity modulates hundreds of genes in *Escherichia coli* (*E. coli*). YmdB, a member of the macrodomain protein family, is one of known trans-acting regulators of RNase III activity; however, the significance of its regulatory role in specific bacterial cellular processes and related genes has not been determined. YmdB overexpression was used to model YmdB-induced RNase III inhibition *in vivo*, and microarray analysis identified gene targets and cellular processes related to RNase III inhibition.

**Results:**

The expression of >2,000 *E. coli* genes was modulated by YmdB induction; 129 genes were strongly regulated, of which 80 have not been reported as RNase III targets. Of these, ten are involved in biofilm formation. Significantly, YmdB overexpression also inhibited biofilm formation via a process that is not uniquely dependent upon RNase III inhibition. Moreover, biofilm formation is interdependently regulated by RpoS, a known stress response regulator and biofilm inhibitor, and by YmdB.

**Conclusions:**

This is the first global profile of target genes modulated by YmdB-induced RNase III inhibition in *E. coli,* and the data reveal a novel, hitherto unrecognized regulatory role for YmdB in biofilm modulation.

## Background

RNase III family members cleave double-stranded RNAs to yield 5′ phosphate and 3′ hydroxyl termini, and are extensively conserved in prokaryotes and eukaryotes [1-7]. During bacterial ribosome biogenesis, RNase III processes the ribosomal RNA (rRNA) precursors [8], and also mediates the maturation and/or degradation of different types of transcripts [9], small RNAs [10,11], and mRNAs containing *rnc*[12,13] or *pnp* genes [14]. The structural and mechanistic features of RNase III have been extensively studied [1-14]; however, questions remain concerning the cellular control of RNase III activity under different physiological conditions.

In *E. coli,* some proteins are known as regulators for endo-RNase activity [15-18]. For example, RraA and RraB negatively regulate RNase E activity [15,16]. In case of RNase III, bacteriophage T7 protein kinase [17] and YmdB [18] identified as an either activator or inhibitor of RNase III function. The activation process by bacteriophage T7 protein kinase is through binding to RNase III and phosphorylates the enzyme on serine [17]. YmdB was the first RNase III-binding inhibitor to be identified *in vivo* using a novel genetic screening approach and, in common with other RNase regulators, YmdB expression is modulated by cold- or growth-stress [18]. YmdB, acting in concert with other uncharacterized stress-mediated trans-acting factors, facilitates the regulation of RNase III activity under growth- [18] or osmotic stress conditions [19]. Several protein identities are proposed for the trans-acting inhibitor(s) and potential targets of their inhibition has been suggested; for example, cellular targets of RNase III activity, such as the RNase III gene itself, *rnc*[12,13]*, pnp*[14]*,* and rRNA processing by YmdB [18] and the level of *bdm* mRNA encoding a protein that promotes biofilm formation by unknown trans-acting factor(s) [19]. The cellular processes required for RNase III inhibition by trans-acting factor(s) during stress responses are unclear; however, one post-transcriptional pathway has been proposed [7], which involves the general stress-responsive regulator, RpoS [20]. By cleaving the *rpoS* mRNA 5′-leader [21], RNase III reduces RpoS production; the presence of YmdB limits this reaction and as a consequence, increases RpoS levels, which supports entry into the stationary phase [7]. This hypothesis behind this process came from a study that used an RNase III mutant [21]; however, to clarify and identify new targets of RNase III inhibition, it is essential to adopt a model that mimics physiological RNase III inhibition via the induction of trans-acting factor(s).

The present study investigated RNase III inhibition via the ectopic expression of the regulatory protein, YmdB, and identified novel targets of inhibition. We also explored the mechanism(s) by which biofilm formation is regulated. Gene expression profiling of the entire *E. coli* open reading frame (ORF) following YmdB overexpression was performed using DNA microarray analysis, and revealed that ~2,000 transcripts were modulated. Of these, 129 genes spanning ten cellular processes were strongly modulated by YmdB expression. About 40 of these were similarly controlled by RNase III, including five novel targets. Moreover, among the YmdB-modulated genes, ten are reported to be related to biofilm formation, the presence of which is a universal feature of bacteria and a component of multicellular communities [22]. Biochemical analyses indicate that induction of YmdB strongly inhibits biofilm formation in a manner similar to that of RpoS, which is a regulator of general stress responses [20] and a biofilm inhibitor [23-25]. Inhibition occurred via two mechanisms that were either dependent or independent of RNase III activity. Genetic studies revealed that the YmdB- and RpoS-induced decrease in biofilm formation required RpoS and YmdB, respectively. In conclusion, we have identified a novel role for YmdB as a modulator of biofilm formation, and revealed how a trans-acting factor can regulate RNase III activity, as well as function independently to enable a rapid response to changing cellular needs.

## Methods

### Bacterial strains, plasmids, primers, and growth conditions

Details of the bacterial strains and plasmids used are given in Additional file 1: Table S1. Primers used for qPCR analysis and DNA sequencing were synthesized by Bioneer (Korea) (Additional file 1: Table S2). All established mutant strains or chromosomal *lacZ* fusions were derived from *E. coli* BW25113. Analysis of *rpoS* promoter activity was based on a plasmid, pKSK001, containing promoter region −92 to +10 of the *rpoS* gene from the *E. coli* K12 genome (GenBank U00096.2) sub-cloned into the *lacZ* transcriptional fusion vector, pSP417 [26], after linearization by *Eco*RI/*Bam*HI. The *lacZ* fusion in pKSK001 was recombined onto the chromosome (KSK003) using the transducing λ phage system, λRS45 [27], via a double recombination event and was verified as previously described [18]. Strain *ΔymdB* was constructed by eliminating the kanamycin cassette (*ymdB::km*^*R*^) from Keio-*ΔymdB* as described previously [28]. Verification of Keio-*ΔymdB*[28], *ΔymdB* (KSK002), Keio-*ΔrpoS*[28], or *rnc14*∙Keio-*ΔrpoS* (KSK005) was carried out by colony PCR using primer pairs *ymdB*-F/-R or *rpoS*-F/-R and Emerald PCR premix (Takara) (Additional file 1: Figure S1), and the PCR products were read by DNA sequencing analysis using the same primers (data not shown). Verification of RNase III mutation was confirmed by Western blot analysis using antibodies against RNase III (Additional file 1: Figure S1). Bacteria were grown in Luria-Bertani (LB) broth or on LB plates at 37°C throughout this study. Antibiotics were used at the following concentrations: kanamycin, 50 μg/mL; tetracycline, 10 μg/mL; and chloramphenicol, 30 μg/mL.

### Microarray analysis

Total RNA was extracted from IPTG (0.1 mM final concentration)-induced *E. coli* BW25113 cells (at an OD_600_ of 1.0) containing either pCA24N (−*gfp*) or ASKA-*ymdB* (−) using an RNeasy® Kit (Qiagen) with two additional DNase treatments. The integrity of the bacterial total RNA was checked by an Agilent 2100 Bioanalyzer. The cDNA probes were prepared by reverse transcription with random priming of total RNA (25 μg) in the presence of aminoallyl-dUTP for 3 h, followed by coupling of probes with Cy3 dye (for the reference) or Cy5 dye (for the test sample) (AP Biotech). The Cy3- or Cy5-labeled cDNA probes were purified, dried, and resuspended in hybridization buffer containing 30% formamide, 5× SSC, 0.1% SDS, and 0.1 mg/mL salmon sperm DNA. The cDNA probes were mixed together and hybridized to customized microarray slides (*E. coli* K12 3 × 15 K microarray; http://www.Mycroarray.com). The image of the slide was scanned with a GenePix 4000B (Axon Instruments, USA) and analyzed by GenePix Pro 3.0 software (Axon Instruments) to obtain the gene expression ratios (reference *vs.* test sample). Microarray data analysis was performed using Genowiz 4.0™ (Ocimum Biosolutions). Global lowess (Locally weighted scatter plot smoothing) method was used for data normalization. The cut-off values for up- or down-regulated genes in duplicate hybridizations were 1.5- or 0.6-fold, respectively.

### RT-qPCR analysis

The *E. coli* strains listed in Additional file 1: Table S1 were grown in LB medium to an OD_600_ of 1.0, and the total RNA was extracted using an RNeasy Mini Kit (Qiagen). Reverse transcription and qPCR (RT-qPCR) analyses were performed using CFX96 (Bio-Rad) with IQ™ SYBR® Green Supermix (Bio-Rad), as described previously [29] and gene specific primers designed by PrimerQuest (http://www.idtdna.com; Additional file 1: Table S2). Primer-dimer and self-complementary formations were checked by melting curve analysis (CFX manager v3.0; Bio-Rad). The 16S rRNA primers were used for normalization [29].

### Crystal violet biofilm assay

The assay was adapted from Nakao *et al.*[30] with the following modifications: *E. coli* were grown in LB broth for 16 h at 37°C and diluted to 5 × 10^6^ CFU/mL in fresh LB broth with or without IPTG. Aliquots (800 μL) dispensed into polystyrene tubes (Falcon 352058, BD Biosciences) and incubated for 24 h at 37°C without shaking. Each data point represents the mean ± standard deviation of ten independent cultures.

### β-galactosidase activity assays

The β-galactosidase activity from whole cells of KSK003 (*λrpoS’-‘lacZ*), KSK004 [SG30013 (*λRpoS750::LacZ*)] [31], RS8872 (*λpnp’-‘lacZ* in *rnc+*) [32], or RS8942 (*λpnp’-‘lacZ* in *rnc14*) [32] overexpressing YmdB from ASKA-*ymdB* (−) was determined as described by Miller [33]. The results are expressed as the means of three independent experiments.

### Protein gel electrophoresis and Western blot analysis

Overexpression of the YmdB and RpoS proteins was detected on Coomassie blue-stained 12% Mini-PROTEAN TGX Precast gels (Bio-Rad). Western blots for RNase III, YmdB, RpoS, or 6x Histidine-tagged YmdB were prepared as described [18], probed with antibodies (1:2,500 dilution) against YmdB, RNase III [18], RpoS (1RS1: Santa Cruz Biotechnology), or 6x Histidine-tagged YmdB (6xHis Epitope Tag Antibody: Thermo Scientific) and developed with Clarity™ western ECL substrate (Bio-Rad). To normalize the signals, antibodies against S1 protein [34] was used as a reference probe (1:100,000 dilution). Anti-rabbit IgG:HRP or anti-mouse IgG:HRP conjugates (Promega; 1:5000 dilution) were used for YmdB/RNase III/S1 proteins or RpoS/6xHistidine tagged YmdB, respectively. Specific proteins were imaged using MyECL and quantified with myImage Analysis software (Thermo Scientific).

## Results

### Analysis of the E. coli transcriptome under conditions mimicking those of an RNase III mutant

To identify which pathways and related genes are mediated by YmdB-modulated RNase III inhibition, a genome-wide analysis of mRNA abundance at single gene resolution was performed. In these experiments, total steady-state RNA extracted from IPTG-induced exponentially grown cells expressing either ASKA-*ymdB* (a part of the ASKA (−) library: a complete set of cloned individual *E. coli* genes encoding proteins with 6x histidines at the N-terminal end and no GFP fusion at the C-terminal end [35]); or pCA24N (a control vector without GFP at the C-terminal end) [29] were analyzed on customized ORF microarray chips. Duplicate arrays were performed with biological replicates to minimize experimental artifacts, and the gene expression profiles of 4,289 genes were averaged and analyzed. YmdB overexpression modulated the relative abundance of more than 2,000 transcripts (data not shown). Of these, 129 genes were strongly regulated (changes in expression of either >1.5 or <0.6 fold) (Additional file 1: Table S3). YmdB was previously identified as an RNase III regulator [18]; thus, we examined the number of YmdB-modulated genes that were related to the down-regulation of RNase III activity to identify RNase III/YmdB-regulatory genes. The microarray data related to YmdB overexpression were compared with the tiling array data for an RNase III mutant [36], in which 592 genes were affected by the absence of RNase III. Of 127 coding genes from the tiling array data, 47 are known RNase III targets and, of these, 37 were similarly regulated by YmdB and the RNase III mutant (Additional file 1: Table S3). This suggests that YmdB modulates these genes by down-regulating RNase III activity. However, 80 genes that were not previously regarded as RNase III targets also appeared to be modulated via an as yet uncharacterized YmdB function(s). When the 80 genes were classified according to the biological process in which they are involved, we identified ten different cellular processes that were modulated by YmdB induction (Table 1). Therefore, the data indicate that YmdB, either as an RNase III regulator or by itself, participates in the regulation of multiple cellular processes within *E. coli*.

**Table 1:** Classification of up- or down-regulated 80 genes when YmdB was overexpressed

**Functions**	**Gene**	**No. of gene**	**Go term ID**
**Transport**	*dppA, emrA, exbB, exuT, fdx, fecI, gutM, icd, mntH, nrfA*^*2*^*, proP, srlA*^*2*^*, srlB*^*2*^*, srlE*^*2*^, *srlR, sucA*^*2*^*, sucC*^*2*^*, sucD*^*2*^*, tdcC, tolB, tolR, yhbE, ynfM*	23	GO:0006810
			GO:0006811
			GO:0006855
			GO:0006865
			GO:0006099
			GO:0009401
			GO:0015031
			GO:0015992
			GO:0017038
			GO:0022900
			GO:0043213
			GO:0055085
**Transcription/replication**	*cspB, cspG, fecI, gutM, lacI, mprA, mukF, mqsR*^3^*, pspB*^1,2^*, pspC*^1,3^*, relE*^3^*, rpoA, rpoB, rpoC, rplD, rpoE*^3^*, rseB, srlR, yoeB, ygiT*^3^	20	GO:0006260
			GO:0006351
			GO:0006352
			GO:0006355
			GO:0045892
			GO:0055072
**Cellular responses**	*cspB, cspG, emrA, mprA, nusA, pspB*^1,3^*, pspC*^1,3^*, pspD*^1,3^*,*	13	GO:0006950
			GO:0009266
			GO:0009271
	*relE*^3^*, rplD, rpoE*^3^*,rseA*^3^*, sseA*		GO:0009408
			GO:0009409
			GO:0046677
**Modification**	*csdA, iscA, iscU, mqsR*^3^*, pheT,*	11	GO:0006432
			GO:0016226
	*relE*^3^*, srlB*^*2*^*, srlE*^*2*^		
			GO:0016310
			GO:0090305
	*ydaL*^3^*, yfhJ, ygdK*		
**Translation**	*mqsR*^3^*, pheT, rplC, rplD, rpsA, rpsJ, yhbC, relE*^3^	8	GO:0006412
			GO:0017148
**Metabolic process**	*fabD, lacI, srlA*^*2*^*, srlB*^*2*^*, srlD*^*2*^*, srlE*^*2*^*, sucA*^*2*^*, ycjM*	8	GO:0008152
**Oxidation-reduction**	*ahpC*^3^*, nrfA*^*2*^*, srlD*^*2*^*, sucA*^*2*^*, torZ, ygjR*	6	GO:0055114
**Biosynthesis**	*fabD*	1	GO:0006633
			GO:0006654
			GO:0008610
**Cell cycle**	*mukF*	1	GO:0007049
			GO:0051301
**Nucleotide binding**	*yeeZ*^3^	1	GO:0000166
			GO:0005524

Genes ^1^up- (>3-fold) or ^2^down-(<0.5-fold) regulated by YmdB overexpression were indicated. Detailed quantitative data are shown in Additional file 1: Table S3.

^3^Gene is related to biofilm formation in literature, even though GO term analysis (http://www.ecocyc.org) did not classify it as such.

### Identification of new RNase III targets regulated by YmdB

Using the results shown in Additional file 1: Table S3, genes that showed the highest changes in expression (>2-fold change) were selected for quantification by real-time quantitative PCR (RT-qPCR). These genes comprised confirmed (*mltD*, *pnp*, *plsX*, and *ahpF*) [14,36] and unconfirmed (*pspA*, *pspB*, *pspC*, *pspD*, and *ahpC*) RNase III targets. For all known RNase III-target genes, increased expression was observed in the RNase III mutant (*rnc14*), which correlated with the YmdB overexpression data (Table 2). Moreover, gene expression decreased or remained at the same level in a *ymdB* knockout strain in which RNase III activity was upregulated, suggesting that YmdB-mediated inhibition of RNase III activity is not involved in the regulation of genes of previously known to be RNase III targets. The abundance of mRNAs for the unconfirmed RNase III target genes was measured in the RNase III mutant and then compared with the data regarding YmdB overexpression (Table 2). From five genes, the expression of the *pspB*, *pspC*, and *ahpC* genes was slightly increased upon both YmdB overexpression and RNase III knockout, further indicating that these genes might be new RNase III targets regulated by YmdB.

**Table 2:** Relative abundance of RNase III-dependent or -independent transcripts by different level of YmdB or RNase III

**RNase III-dependent genes**	**Microarray**^**1**^	**qPCR-***Δ* ***ymdB***^**2**^	**qPCR-YmdB**^**3**^	**qPCR-*rnc14***^**4**^
*mltD*	3.66	3.06 ± 0.04	7.37 ± 0.03	39.80 ± 0.01
*PnP*	3.06	0.84 ± 0.01	3.27 ± 0.36	8.02 ± 0.02
*plsX*	3.01	2.98 ± 0.01	2.86 ± 0.31	21.37 ± 0.01
*ahpF*	2.48	0.90 ± 0.02	3.34 ± 0.33	7.72 ± 0.01
*yhdE*	2.26	1.90 ± 0.01	2.37 ± 0.20	3.93 ± 0.01
**RNase III-independent genes**	**Microarray**^**1**^	**qPCR-***Δ* ***ymdB***^**2**^	**qPCR-YmdB**^**3**^	**qPCR-*rnc14***^**4**^
*pspB*	5.18	0.88 ± 0.13	1.53 ± 0.01	1.36 ± 0.01
*pspA*	4.46	0.78 ± 0.01	1.50 ± 0.01	1.15 ± 0.01
*pspD*	4.30	0.82 ± 0.01	2.45 ± 0.06	1.86 ± 0.02
*pspC*	3.86	1.01 ± 0.01	1.59 ± 0.02	1.38 ± 0.02
*ahpC*	2.81	0.67 ± 0.01	3.73 ± 0.01	3.30 ± 0.01

^1^Fold-change of each transcript levels from microarray analysis (Additional file 1: Table S3): YmdB overexpression from ASKA-*ymdB*(−) *vs* pCA24N (−*gfp*) in wild-type (BW25113) background.

Relative ratios of each transcript levels determined by qPCR with specific primers (Additional file 1: Table S2) are indicated: ^2^*ymdB* knockout (*ΔymdB*: KSK002) *vs* BW25113 (*ymdB*), ^3^YmdB overexpression from ASKA-*ymdB* (−) *vs* pCA24N (−*gfp*) or ^4^RNase III mutant (*rnc14*:KSK001) *vs* BW25113 *(rnc+).*

### Identification of YmdB as a protein that inhibits biofilm formation

**Figure 1:**
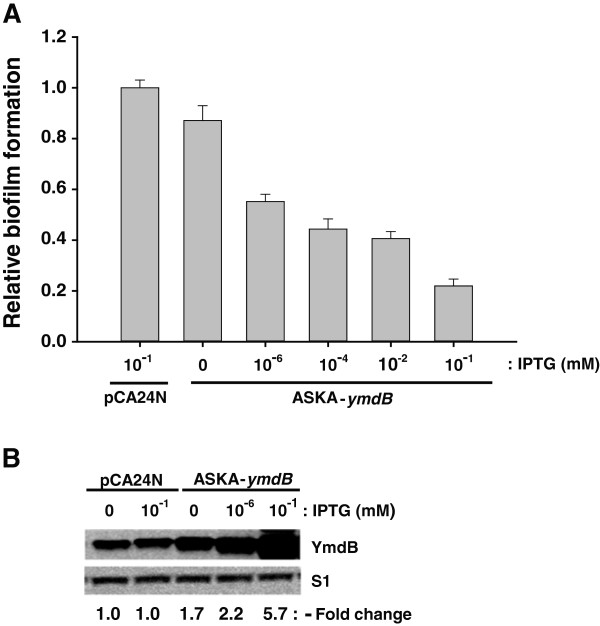
**YmdB inhibits biofilm formation. (A)** Inhibition of biofilm formation by YmdB overexpression. *E. coli* cells containing either pCA24N (−*gfp*) or ASKA-*ymdB* (−) were grown at 37°C for 24 h in LB medium containing 0 to 10^-1^ mM IPTG. Biofilm formation was analyzed, and mean values calculated (n = 10, p = 0.05). **(B)** Western blot analysis of YmdB expression levels. Total cellular proteins from the cells described in **(A)** were reacted with antibodies against YmdB or S1. Changes in YmdB protein levels from ASKA-*ymdB* induced by IPTG were determined relative to the levels of chromosomally-encoded YmdB protein derived from pCA24N *(-gfp)* vector-containing cells (indicated below).

The results obtained thus far suggest a role for YmdB in biofilm synthesis. Ten genes related to biofilm formation [37-40] were modulated by YmdB overexpression (Table 1); in particular, genes induced within the biofilm were strongly upregulated, including *rpoE*[41] and *pspABCDE*[41,42]. Additionally, *rpoS* and *bdm*, both known targets of RNase III and related to either the down- or up-regulation of biofilm formation [19,21,36], were upregulated (by ~1.5- and 1.8-fold, respectively). To investigate this further, biofilm formation by BW25113 cells containing either pCA24N (−*gfp*) or ASKA-*ymdB* (−) was measured after 24 h of growth without agitation in LB containing different concentrations of IPTG (0 to 0.1 mM). Compared with cells harboring the control vector, increased expression of YmdB inhibited biofilm formation by up to 80% in the presence or absence of IPTG (Figure 1A). The effect was linearly correlated with IPTG concentration (10^-6^ to 10^-1^ mM), with no further reduction observed above 1 mM IPTG (data not shown). The physiological relevance of this inhibitory effect of YmdB is shown in Figure 1B. The total relative level of YmdB protein was assessed by Western Blotting [18] and was found to increase by 2.2- to 5.7-fold in the presence of increasing concentrations of IPTG (Figure 1B) (increased YmdB expression in the absence of IPTG results from the leaky *lac* promoter in the ASKA plasmids) [35]. The changes in YmdB levels are similar to those observed *in vivo* following growth- or cold-stress (2- to 8-fold) [18] and are thus physiologically relevant.

### RNase III does not affect biofilm inhibition by YmdB

**Figure 2:**
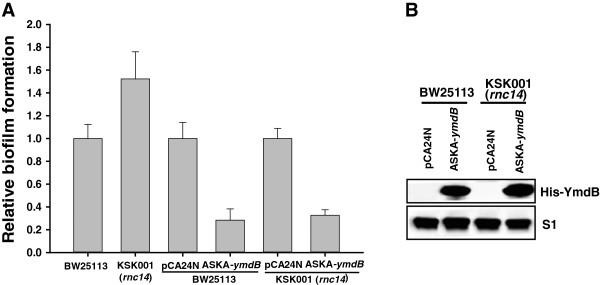
**YmdB inhibits biofilm formation in an RNase III-independent manner. (A)** Effect of the presence or absence of RNase III on YmdB-mediated inhibition of biofilm formation. Biofilm formation by BW25113 (*rnc+*) or KSK001 (*rnc14*) cells with or without plasmid [pCA24N (−*gfp*) or ASKA-*ymdB* (−)] was measured using cells grown at 37°C for 24 h in LB medium containing IPTG (0.1 mM final) Mean values (n = 10, p = 0.05) are shown. “Relative biofilm formation” for KSK001 and ASKA-*ymdB* (in BW25113 or KSK001) was determined relative to the biofilm formation by each control set (BW25113 or pCA24N; set to 1.0). **(B)** Expression levels of YmdB. The expression of YmdB (His-YmdB) in total cell lysates **(from A)** was detected by immunoblotting with 6xHis Epitope Tag antibody as described in Methods. S1 protein level was used as loading control.

The YmdB protein regulates RNase III activity through the formation through the proposed formation of an RNase III/YmdB heterocomplex [18]; hence, it was important to clarify whether the biofilm phenotype mediated by ectopic expression of YmdB is similar to that mediated by RNase III inhibition. Biofilm formation in the absence of RNase III (*rnc14*) increased by ~52% (Figure 2A), implying that inhibition of biofilm formation is independent of RNase III/YmdB heterocomplex, and that an alternative, hitherto uncharacterized, function of YmdB exists. To verify this possibility, we measured the inhibition of biofilm formation in the presence of YmdB overexpression (confirmed in Figure 2B) against an *rnc +* and *rnc14* background. The results showed that inhibition was almost the same between the wild-type strain (~70%) and the strain lacking RNase III (~67%) (Figure 2A). By contrast, processing of *pnp’-‘lacZ* mRNA, a known target for YmdB-mediated inhibition of RNase III activity [18], is fully dependent on RNase III (Additional file 1: Figure S2). Taken together, these results indicate that YmdB overexpression inhibits biofilm formation via an RNase III-independent pathway.

### RpoS is required for the inhibition of biofilm formation by YmdB

**Figure 3:**
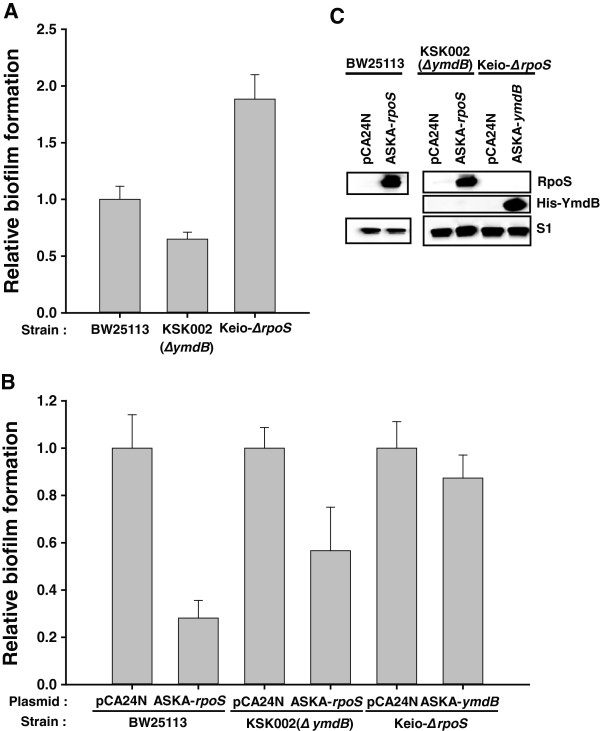
**Interdependency on YmdB and RpoS for biofilm formation. (A)** Effect of knocking out *ymdB* or *rpoS* on biofilm formation. Biofilm formation was measured in wild-type (*ymdB +* or *rpoS+*), KSK002 (*∆ymdB*) and *rpoS* mutant (Keio-*∆rpoS*) cells. **(B)** Dependency of RpoS and YmdB phenotype on biofilm formation. The effect of ectopic expression of RpoS or YmdB in the absence of *ymdB* or *rpoS,* respectively*,* on biofilm formation was determined. **(C)** Expression of RpoS and YmdB. Protein expression was detected by immunoblotting using antibodies against RpoS and 6xHistidine tagged YmdB (His-YmdB) as described in Methods. S1 protein level was used as a loading control. All biofilm formation data were obtained as described in Methods. Data represent the mean values from ten independent experiments.

While it was clear that YmdB induction decreased biofilm formation (Figure 1), biofilm formation also decreased by ~ 35% in the absence of *ymdB* (*ΔymdB*) gene in the chromosome (Figure 3A). This could indicate that YmdB is involved in, but not essential for, the inhibition of biofilm formation in *E. coli,* or that increased levels of YmdB affect biofilm formation by modulating associated cellular proteins and their pathways. To test this hypothesis, we sought to identify candidate genes whose mRNA levels were increased by YmdB (Table 1) and which have a known effect on the biofilm phenotype. One strong candidate is RpoS, a stress-responsive sigma factor [21], which when overexpressed led to a reduction in biofilm formation (Figures 3B,C; [25]). To determine whether YmdB-mediated inhibition of biofilm formation is dependent on the presence or absence of *rpoS,* we measured biofilm formation in an *rpoS* knockout strain (Keio-*ΔrpoS*). Biofilm formation was activated in the *rpoS* knockout (Figures 3A,C). Subsequent introduction of a plasmid overexpressing YmdB only decreased biofilm inhibition by 12% in the *rpoS* knockout (Figure 3B) whereas it resulted in 70% inhibition in wild-type cells (Figure 2A); thus, the inhibition of biofilm formation by YmdB is RpoS-dependent.

### Both YmdB and RpoS interdependently regulate gene expression and activity on biofilm formation

**Figure 4:**
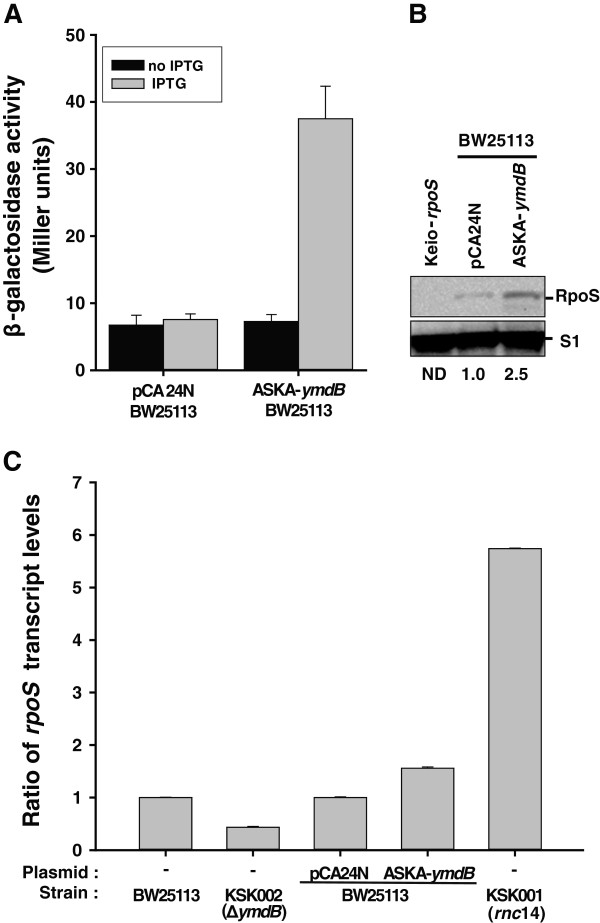
**Regulation of RpoS levels and activity by YmdB. (A)** Effect of YmdB on *in vivo* expression levels of RpoS. KS004 [SG30013 (*λRpoS750::LacZ*] [31] strains containing either pCA24N (−*gfp*) or ASKA-*ymdB* (−) were grown to OD_600_ = 0.2, induced by IPTG (0.1 mM final), and further grown to OD_600_ = 1.0. Aliquots were then assayed for β-galactosidase activity. Data represent the mean values from n = 3 experiments (p = 0.05). **(B)** Expression level of RpoS. Total lysates prepared from the cell described in **(A)** and from Keio-*∆rpoS* cells were immunoblotted antibodies against RpoS and S1. The Keio-*∆rpoS* strain is included to show the specificity of the antibody. The relative levels of RpoS normalized against S1 protein are shown. ND, not determined. **(C)** Determination of steady-state levels of *rpoS* transcript induced by YmdB. cDNA synthesized from total RNA obtained from BW25113, KSK002 (*∆ymdB*), KSK001 (*rnc14*) or BW25113 cells containing either pCA24N (−*gfp*) or ASKA-*ymdB* (−) were qPCR amplified using the *rpoS*- or 16S RNA-specific primer sets listed in Additional file 1: Table S2 and then compared. Data represent the mean values from triplicate experiments.

Since YmdB is transcriptionally activated by RpoS [18] and the level of *rpoS* transcripts was increased by YmdB overexpression, it is possible that YmdB modulates RpoS expression. YmdB could affect this change in *rpoS* transcript levels by either acting as an as yet unknown transcription factor or by acting as an effector protein for the factor(s) involved in *rpoS* transcription. We found that YmdB overexpression had no effect on *rpoS* promoter activity (data not shown), thereby excluding any role as a transcription factor. A linear relationship between *rpoS* transcript levels and RpoS protein levels was then investigated following YmdB induction, and similar increases (~2.5-fold) in the induced β-galactosidase activity of the *rpoS’-‘lacZ* protein fusion and the RpoS protein level were observed (Figures 4A,B). Moreover, the steady-state level of *rpoS* transcript (Figure 4C) was oppositely regulated in the absence of chromosomal *ymdB*. Additionally, the level of *rpoS* transcript following YmdB overexpression was lower than that in the RNase III mutant strain. These data suggest that YmdB-mediated regulation of RNase III activity alone cannot fully regulate the processing of the 5′ UTR of *rpoS* mRNA. Because RpoS can negatively regulate biofilm formation by itself (Figure 3B) and is also required for complete YmdB function (Figure 3B), it is a matter of debate whether YmdB can modulate RpoS activity. When the RpoS protein was overexpressed in a wild-type and in an *ymdB* knockout strain, RpoS-mediated inhibition of biofilm formation was decreased from 70% to 43% (Figure 3B). This, when taken together with the other data, suggests that the regulation of RpoS function during biofilm formation is dependent upon YmdB. Moreover, RpoS overexpression phenotype on biofilm inhibition was not dependent upon the presence of RNase III activity (Additional file 1: Figure S3). Thus, YmdB is a novel post-transcriptional regulator of RpoS levels that acts independently of RNase III.

## Discussion

**Figure 5:**
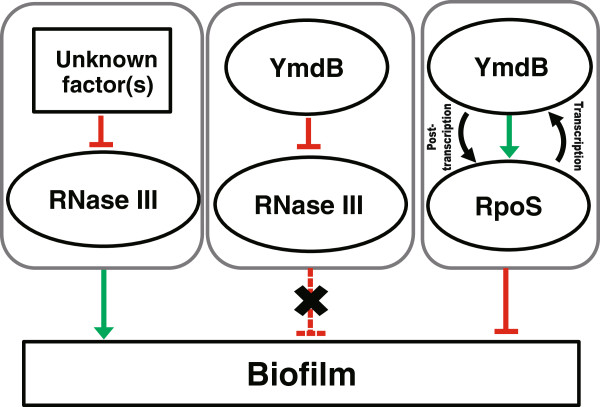
**A schematic model of biofilm formation and gene expression involving YmdB, RpoS, and RNase III*.*** Two different pathways for biofilm formation are proposed: an RNase III-dependent pathway in which other uncharacterized factor(s) inhibit RNase III activity, thereby upregulating biofilm formation, and an RNase III-independent pathway in which both YmdB and RpoS interdependently regulate the inhibition of biofilm formation. In terms of gene expression, the level of RpoS is post-transcriptionally regulated by YmdB either directly or indirectly via the inhibition of RNase III activity [18,20], while the level of YmdB is regulated transcriptionally by the RpoS protein [18].

The results presented herein demonstrate that YmdB is a major regulator of RNase III activity in *E. coli*, modulating more than 30% of the genes targeted by RNase III. In addition, the results of a microarray analysis following YmdB overexpression (which identified changes in biofilm-related genes and a decrease in biofilm formation) indicate a novel role for YmdB as a modulator of biofilm formation. Previous results indicated that overexpression of RpoS was associated with decreased biofilm formation [25]. Our microarray, qPCR, and Western blotting data showed that overexpression of YmdB increased the levels of RpoS (Additional file 1: Tables S3, Figures 2, 3 and 4). Moreover, YmdB modulated RpoS levels and activity of biofilm formation (Figures 3, 4). Thus, we propose a model to illustrate the multiple roles played by YmdB during gene expression and biofilm formation (Figure 5).

The 5′ UTR of *rpoS* mRNA is a known target of RNase III and its levels increase when RNase III activity is ablated [21]. Because biofilm formation is influenced by RpoS levels, it may be proposed that the *rpoS* mRNA is responsive to YmdB-directed RNase III inhibition. However, this is not the case because the decrease in biofilm formation following YmdB expression was not reversed in the absence of RNase III (Figure 2), suggesting that regulation of RNase III activity by YmdB is not essential for the inhibition of biofilm formation. Thus, the major mechanism underlying biofilm regulation by YmdB appears to be RNase III-independent (Figure 5).

A screen of potential regulatory gene(s) with a YmdB-mediated phenotype demonstrated that RpoS is necessary for inhibiting biofilm formation (Figure 3); RpoS activates the transcription of *ymdB*[18]; thus, it is highly plausible that the RpoS gene is an upstream regulator of YmdB transcription and the resultant phenotypes. Conversely, the possibility that YmdB is a transcription factor that activates *rpoS* transcription was initially suggested by observations that RpoS levels were increased by YmdB overexpression, and that YmdB and RpoS are both required for the decrease in biofilm formation. However, this theory was rejected because increases in YmdB expression had no effect on promoter activity (data not shown). Hence, YmdB-induced modulation of RpoS levels must occur via post-transcriptional regulation (Figure 4). It is also possible that YmdB modulates other *rpoS* transcription factor(s), although we have not identified which other transcription factors are required for this response. Overall, the data suggest that YmdB and RpoS are co-regulators of biofilm formation (Figure 5).

The identification of a novel role for YmdB is not altogether surprising, since eukaryotic macrodomain proteins can have multiple roles [43,44], and YmdB has additional functions in bacteria [45,46]. For instance, in *E. coli* YmdB deacetylates the sirtuin product of O-acetyl-ADP-ribose and reforms ADP ribose [45]. The present study reveals that YmdB modulates the expression of genes involved in physiologically important pathways (Table 1); hence, YmdB could act as a general regulator in a variety of cellular processes. Further examination of such a potential role for YmdB and its family members in bacteria is necessary. YmdB is also required to be coexpressed for the complementation of a function of ClsC, a recently identified cardiolipin synthase in *E. coli*[45]. ClsC utilizes phosphatidylethanolamines (PE) as the phosphatidyl donor to phosphatidylglycine (PG) to form cardiolipin (CL) [46]. While YmdB is apparently not a direct modulator of that pathway (since changes in *clsC* (*ymdC*) gene expression in the microarrays were negligible (a 1.1-fold increase only); (data not shown), it may modulate it indirectly via the action of the fatty acid biosynthesis gene, *fabD* (Table 1), on the CL synthesis-regulating gene; however, such a role has not been confirmed.

The ectopic expression of YmdB almost completely regulates RNase III activity with respect to several targets, including *pnp*, *rnc* and ribosomal RNA processing (Additional file 1: Figure S2) [6]; however, biofilm formation is not solely dependent upon YmdB-directed RNase III regulation, suggesting that gene expression data will be useful for identifying unknown RNase III-independently regulated YmdB functions.

Several trans-acting factors that modulate the RNase activity of both exo- and endo-RNases have been identified in *E. coli*[15-18,47,48]. Among these four trans-acting regulatory proteins for endo-RNase activity have been well characterized in *E. coli*: RraA [15] and RraB [16] for RNase E, and bacteriophage T7 protein kinase [17] and YmdB [18] for RNase III. The presence of homologs in other species suggests such regulation of endo-RNase activity is generally required for bacterial physiology. Recently, gene expression profiling revealed a role for RraA in regulating the SOS response, a mechanism which responds to the stress caused by DNA damage [15,49]. RNase III modulates approximately 12% (592 genes) of the *E. coli* genome [35]; using YmdB-mediated down-regulation of RNase III rather than an RNase III mutant retains the ability to measure the effect of trans-acting factor(s) and hence the correct physiological modulation of RNase III. Because YmdB regulates the turnover of approximately 30% of the target genes of RNase III (Additional file 1: Table S3) and the *rpoS* level is not completely regulated by YmdB (Figure 4), either other regulator(s) that result RNase III mutant-like conditions must be present or YmdB partially regulates the physiology of the RNase III-mutant to induce the up-regulation of an RNase III activator that has yet to be identified.

## Conclusions

The data presented herein show that YmdB functions both to regulate RNase III activity and to modulate bacterial biofilm formation; therefore, YmdB seems to be a multifunctional bacterial macrodomain protein, similar to that in eukaryotic cells. Furthermore, this protein will make it possible to design a more intelligent synthetic scaffold for producing bacterial cells that modulate difficult-to-treat pathogens that depend upon biofilm production.

### Availability of supporting data

The data sets supporting the results of this article are included within the article and in Additional file 1.

## Competing interests

The authors declare that they have no competing interest.

## Authors’ contributions

TYK, JYL, and KSK conceived of and designed all the experiments in the paper, executed experiments, collected, and interpreted the data, and drafted the manuscript. All authors read and approved the final manuscript.

## Supplementary Material

Additional file 1: Table S1 Strains and plasmids used in this study. **Table S2.** List of primers used in this study. **Table S3.** Differential gene expression profiles of *E. coli* 129 genes. **Figure S1.** Verification of *rpoS*, *ymdB*, and *rnc* mutants. PCR validation of (A) Keio-*∆rpoS* or (B) Keio*-∆ymdB* and *∆ymdB*. (C) Schematic representations of PCR regions. (D) Western-blot analysis verifying RNase III mutation. **Figure S2.** Dependency of YmdB-mediated down-regulation of RNase III activity upon the presence of RNase III. **Figure S3.** Interdependency of RpoS and RNase III for biofilm formation. **Figure S4.** Dependency of YmdB-mediated phenotype upon the absence of RpoS and RNase III. (A) Effect of biofilm formation by double mutation of RpoS and RNase III. (B) Effect of YmdB-mediated inhibition of biofilm formation in double mutation of RNase III and RpoS. Click here for file
