# Supplementary material for: Escherichia coli YmdB regulates biofilm formation independently of its role as an RNase III modulator
Source: BMC Microbiol. 2013 Nov 24;13:266. doi: 10.1186/1471-2180-13-266 (PMC4222554; doi:10.1186/1471-2180-13-266)
Supplement: Additional file 1: Table S1 — Strains and plasmids used in this study. Table S2. List of primers used in this study. Table S3. Differential gene expression profiles of E. coli 129 genes. Figure S1. Verification of rpoS, ymdB, and rnc mutants. PCR validation of (A) Keio-∆rpoS or (B) Keio-∆ymdB and ∆ymdB. (C) Schematic representations of PCR regions. (D) Western-blot analysis verifying RNase III mutation. Figure S2. Dependency of YmdB-mediated down-regulation of RNase III activity upon the presence of RNase III. Figure S3. Interdependency of RpoS and RNase III for biofilm formation. Figure S4. Dependency of YmdB-mediated phenotype upon the absence of RpoS and RNase III. (A) Effect of biofilm formation by double mutation of RpoS and RNase III. (B) Effect of YmdB-mediated inhibition of biofilm formation in double mutation of RNase III and RpoS. [file 1471-2180-13-266-S1.pdf]

Table S1. Strains and plasmids used in this study

Strains and plasmids	Genotype	Description and References
**Strains**		
BW25113	*rrnB*$_{T14}$ Δ*lacZ*$_{WJ16}$*hsdR*514 Δ*araBAD*$_{AH33}$ Δ*rhaBAD*$_{LD78}$	Wild-type strain [28]
KSK001	BW25113 (*rnc14::Tn10*)	[29]
KSK002	BW25113 (*ΔymdB*) (-Km^R)	Elimination of Km^R cassette from Keio-*ΔymdB* (JW1032) [28]; This study
KSK003	BW25113 (λSG30013)	Transcription, translation, and degradation of RpoS [31]
KSK004	BW25113 (λ*rpoS'*-'*lacZ*)	This study
KSK005	BW25113 (*rnc14*·Keio-*ΔrpoS*)	This study
Keio-*ΔrpoS*	Keio collection; JW5437	[28]
RS8872	P90C *pnp'*-'*lacZ*	[32]
RS8942	P90C *rnc14::Tn10 pnp'*-'*lacZ*	[32]
pCA24N (-*gfp*)	LacI^q, Cm^{R,} -*gfp*	[29]
**Plasmids**		
ASKA-*ymdB* (-)	ASKA (-) collection	[35]
ASKA-*rpoS* (-)	ASKA (-) collection	[35]
pSP417	Cloning vector with a promoterless lacZ	[26]
pKSK001	pSP417-*rpoS* (-92 to +10)	*rpoS* transcriptional fusion: *rpoS*'-'*lacZ*; This study

Table S2. List of primers used in this study

qPCR analysis		
**Primer**	**Forward (5’ to 3’)**	**Reverse (5’ to 3’)**
**mltD**	TTC ATT GGC GAC GAG CTA AA	ACA TCG TGG AGA TAG CTC TTA TTG
**pnp**	CGA TAC CTT CCT GTT CCA CTA C	AGA CGA CCG TGA CCA ATT TC
**plsX**	TTC CTG CGC AGT CAG TTA TC	TTG CGC TCG ACC TTC TTT
**ahpF**	CAA GGA ACT GTT GGC TGA AAT C	TGG GTT GGT GAT CAG GAA AG
**yhdE**	TCT GTA TTT AGC TTC CGG TTC TC	CTC CTC AAT GCC CGT AAC AAT A
**pspB**	AGT GAG CAG CAG CGA TTA G	CCT CCA GTT CGG ATG TTC TG
**pspA**	GAT GAA GCA ATG GCT CGT TTC	AAC TGA TCG TCC AGC GAT TT
**pspD**	AGG CAA GCT GGT ACT TCT TAC	TCG GCG AGC AAC TGA TTT
**pspC**	TGG CGG GCA TTA ATC TCA ATA	CCA GGA TAC GCA CCA GTT T
**ahpC**	CTG GAG CGT CTT CTT CTT CTA C	GTA TAC GTC TAC GCC CAG TTT C
**rpoS**	CAGAGCATCGTCAAATGGCTGTT	ATCTTCCAGTGTTGCCGCTTCGTA
**16S**	GCT ACA ATG GCG CAT ACA AA	TTC ATG GAG TCG AGT TGC AG

DNA sequencing, colony PCR, or cloning		
**Primer**	**Sequence (5’ to 3’)**	**Purpose**
**pCA24N_F**	GAT AAC AAT TTC ACA CAG AAT TCA TTA AAG AG	Confirmation of ASKA clones
**pSP417-rpoS_F**	G**GA ATT C**GC CGA AAT GCC ATT ACC	Construction of *rpoS* transcriptional fusion
**pSP417-rpoS_R**	CG**G GAT CC**T TCA CCC GAA GAC TC	Construction of *rpoS* transcriptional fusion
**LacZ+139**	GGG CCT CTT CGC TAT TAC GC	Sequencing of pSP417-*rpoS*
**rpoS_F**	CCC ATA ACG ACA CAA TGC TG	Confirmation of Keio-*rpoS*
**rpoS_R**	TAT CTG GGG TTG TCG GTA GC	Confirmation of Keio-*rpoS*
**ymdB_F**	CAA GAA GGT GTA AGG AGG CGT	Confirmation of Keio-*ymdB* or *ΔymdB*
**ymdB_R**	GCG TAT GCT CTG CCA TTT CG	Confirmation of Keio-*ymdB* or *ΔymdB*

Underlined sequences are restriction enzyme sites.

Table S3. Differential gene expression profiles of 129 *E. coli* genes. Genes showing a >1.5- or <0.6-fold change in expression in YmdB overexpressing cells are listed. Known RNase III targets from tiling array analysis [34] are marked as either purple (indicating a correlation with microarray data), or green (no correlation) [34]. Customized microarray IDs, HCL (hierarchical clustering) numbers, locus tag numbers, and gene product information are indicated.

Custom ID	HCL	Gene	Locus_tag	ASKA-*ymdB* / pCA24N	P value	Gene Product
825982	19	*pspB*	b1305	5.18	0.00	DNA-binding transcriptional regulator of psp
825984	22	*pspA*	b1304	4.46	0.01	regulatory protein for phage-shock-protein
825995	21	*pspD*	b1307	4.30	0.00	peripheral inner membrane phage-shock protein
825997	23	*pspC*	b1306	3.86	0.01	DNA-binding transcriptional activator
822955	24	***mltD***	**b0211**	3.66	0.01	predicted membrane-bound lytic murein
831149	16	***pnp***	**b3164**	3.06	0.01	polynucleotide phosphorylase/polyadenylase
825379	17	***plsX***	**b1090**	3.01	0.00	fatty acid/phospholipid synthesis protein
824049	18	*ahpC*	b0605	2.81	0.00	alkyl hydroperoxide reductase, C22 subunit
824058	14	***ahpF***	**b0606**	2.48	0.02	alkyl hydroperoxide reductase, F52a subunit,
827136	12	***pheS***	**b1714**	2.34	0.01	phenylalanine tRNA synthetase, alpha subunit
830188	8	*ygdI*	b2809	2.29	0.01	predicted protein
829604	11	*rpoE*	b2573	2.28	0.02	RNA polymerase, sigma 24 (sigma E) factor
834457	7	***fhuF***	**b4367**	2.27	0.01	ferric iron reductase involved in ferric
831414	10	***yhdE***	**b3248**	2.26	0.03	conserved protein
831390	9	***rng***	**b3247**	2.25	0.02	ribonuclease G
831138	2	***nlpI***	**b3163**	2.24	0.01	conserved protein
826800	3	*ynfM*	b1596	2.19	0.01	predicted transporter
830754	5	*mqsR*	b3022	2.15	0.00	predicted cyanide hydratase
829565	1	***rnc***	**b2567**	2.15	0.02	RNase III
825385	6	***fabH***	**b1091**	2.13	0.01	3-oxoacyl-[acyl-carrier-protein] synthase III
829894	4	*emrA*	b2685	2.12	0.02	multidrug efflux system
834252	37	*fecI*	b4293	2.06	0.02	KpLE2 phage-like element; RNA polymerase, sigma
829418	38	***hisS***	**b2514**	2.06	0.01	histidyl tRNA synthetase
822946	42	***dnaE***	**b0184**	2.01	0.04	DNA polymerase III alpha subunit
827292	41	*pheT*	b1713	2.01	0.00	phenylalanine tRNA synthetase, beta subunit
830742	40	*exbB*	b3006	2.01	0.00	membrane spanning protein in TonB-ExbB-ExbD
830220	39	*csdA*	b2810	1.98	0.00	cysteine sulfinate desulfinase
825109	26	*cspG*	b0990	1.94	0.03	DNA-binding transcriptional regulator
824473	30	***tolA***	**b0739**	1.93	0.01	membrane anchored protein in TolA-TolQ-TolR
824785	29	*clpS*	b0881	1.92	0.01	regulatory protein for ClpA substrate
833492	28	*rpoC*	b3988	1.90	0.00	RNA polymerase, beta prime subunit

834473	27	*yjjZ*	b4567	1.90	0.01	predicted protein
829491	33	***hscB***	**b2527**	1.89	0.02	DnaJ-like molecular chaperone specific for IscU
829583	31	*rseB*	b2571	1.88	0.04	anti-sigma factor
833418	32	*rpoB*	b3987	1.87	0.02	RNA polymerase, beta subunit
827102	34	***pps***	**b1702**	1.87	0.00	phosphoenolpyruvate synthase
829465	35	***hscA***	**b2526**	1.83	0.01	DnaK-like molecular chaperone specific for IscU
823328	36	*lacZ*	b0344	1.83	0.02	beta-D-galactosidase
829610	45	*rseA*	b2572	1.79	0.04	anti-sigma factor
825414	44	*fabD*	b1092	1.79	0.03	malonyl-CoA-[acyl-carrier-protein] transacylase
826526	43	*bdm*	b1481	1.78	0.03	biofilm-dependent modulation protein
831205	56	*yhbE*	b3184	1.75	0.01	conserved inner membrane protein
829176	46	***cysU***	**b2424**	1.75	0.05	sulfate/thiosulfate transporter subunit
827952	47	*yoeB*	b4539	1.75	0.02	toxin of the YoeB-YefM toxin-antitoxin system
827610	48	*torZ*	b1872	1.74	0.03	trimethylamine N-oxide reductase system III,
826101	57	*ydaL*	b1340	1.73	0.01	conserved protein
822817	58	***fhuA***	**b0150**	1.72	0.00	ferrichrome outer membrane transporter
824008	55	***entE***	**b0594**	1.72	0.02	2,3-dihydroxybenzoate-AMP ligase component of
831573	54	*rplC*	b3320	1.71	0.02	50S ribosomal subunit protein L3
830208	53	*ygdK*	b2811	1.70	0.03	predicted Fe-S metabolism protein
826315	61	*ydbA*	b1405	1.70	0.04	predicted outer membrane protein, C-ter fragment
826704	52	*relE*	b1563	1.70	0.01	Qin prophage; toxin of the RelE-RelB
831594	49	*rplD*	b3319	1.68	0.02	50S ribosomal subunit protein L4
831167	60	*nusA*	b3169	1.68	0.03	transcription termination/antitermination L
829065	59	*ypeC*	b2390	1.68	0.05	conserved protein
829568	50	*rseC*	b2570	1.68	0.01	RseC protein involved in reduction of the SoxR
829453	51	*fdx*	b2525	1.67	0.01	[2Fe-2S] ferredoxin
829068	68	*mntH*	b2392	1.65	0.03	manganese/divalent cation transporter
829398	71	*yfgJ*	b2510	1.65	0.01	predicted protein
826698	62	*cspB*	b1557	1.65	0.03	Qin prophage; cold shock protein
824907	69	*mukF*	b0922	1.64	0.03	Involved in chromosome partioning, Ca2+ binding
824394	74	*tolR*	b0738	1.64	0.01	membrane spanning protein in TolA-TolQ-TolR
828387	67	***cirA***	**b2155**	1.64	0.03	ferric iron-catecholate outer membrane
829439	73	*yfhJ*	b2524	1.64	0.01	conserved protein
829456	64	*iscA*	b2528	1.64	0.06	FeS cluster assembly protein
827946	72	***yeeE***	**b2013**	1.64	0.00	predicted inner membrane protein
822625	82	***ftsQ***	**b0093**	1.63	0.00	membrane anchored protein involved in growth of
824002	63	***entC***	**b0593**	1.62	0.04	isochorismate synthase 1
824883	70	*rpsA*	b0911	1.62	0.02	30S ribosomal subunit protein S1
831576	79	***rpoA***	**b3295**	1.61	0.00	RNA polymerase, alpha subunit
826000	81	*ycjM*	b1309	1.61	0.01	predicted glucosyltransferase

831164	80	*yhbC*	b3170	1.61	0.00	conserved protein
824996	66	*pqiB*	b0951	1.61	0.03	paraquat-inducible protein B
830244	77	*ptr*	b2821	1.60	0.01	protease III
829050	25	*yfdM*	b2356	1.60	0.20	CPS-53 (KpLE1) prophage; predicted
831472	76	*yhdL*	b4550	1.60	0.01	conserved protein
833839	65	*proP*	b4111	1.59	0.03	proline/glycine betaine transporter
831495	75	***rpsK***	**b3297**	1.59	0.00	30S ribosomal subunit protein S11
823974	78	***fepA***	**b0584**	1.59	0.02	iron-enterobactin outer membrane transporter
829571	93	*iscU*	b2529	1.55	0.03	scaffold protein
830028	85	*truD*	b2745	1.54	0.02	pseudoruidine synthase
832252	84	***bax***	**b3570**	1.54	0.04	conserved protein
829589	89	***lepA***	**b2569**	1.52	0.03	GTP-binding membrane protein
831579	87	*rpsJ*	b3321	1.52	0.01	30S ribosomal subunit protein S10
829430	94	***yfgM***	**b2513**	1.52	0.01	conserved protein
829194	86	*yfeS*	b2420	1.52	0.02	conserved protein
828005	88	*yeeZ*	b2016	1.52	0.01	predicted epimerase, with NAD(P)-binding
825796	95	*ychS*	b1228	1.51	0.02	predicted protein
826886	90	***rsxA***	**b1627**	1.51	0.04	predicted inner membrane subunit
824397	100	*tolB*	b0740	1.51	0.01	periplasmic protein
830751	99	*ygiT*	b3021	1.51	0.01	predicted DNA-binding transcriptional regulator
823314	92	*lacI*	b0345	1.50	0.03	DNA-binding transcriptional repressor
831873	2453	*glgC*	b3430	0.60	0.01	glucose-1-phosphate adenylyltransferase
822826	2466	*sfsA*	b0146	0.60	0.05	predicted DNA-binding transcriptional regulator
825036	2454	***pflB***	**b0903**	0.59	0.00	pyruvate formate lyase I
832662	2449	***tnaA***	**b3708**	0.59	0.01	tryptophanase/L-cysteine desulfhydrase,
824169	2464	*ybeL*	b0643	0.59	0.04	conserved protein
833534	2450	***malE***	**b4034**	0.59	0.01	maltose transporter subunit
829912	2451	*srlR*	b2707	0.59	0.00	DNA-bindng transcriptional repressor
829427	2448	*sseA*	b2521	0.59	0.04	3-mercaptopyruvate sulfurtransferase
829667	2421	***clpB***	**b2592**	0.58	0.03	protein disaggregation chaperone
825513	2422	*icd*	b1136	0.58	0.01	e14 prophage; isocitrate dehydrogenase, specific
830994	2419	*exuT*	b3093	0.57	0.04	hexuronate transporter
829906	2420	*gutM*	b2706	0.56	0.02	DNA-binding transcriptional activator of
827411	2418	***manX***	**b1817**	0.55	0.00	fused mannose-specific PTS enzymes: IIA
831841	2417	***malP***	**b3417**	0.55	0.01	maltodextrin phosphorylase
830921	2413	*ygjR*	b3087	0.55	0.02	predicted NAD(P)-binding dehydrogenase
830730	2416	***hybO***	**b2997**	0.54	0.02	hydrogenase 2, small subunit
831040	2415	*tdcC*	b3116	0.54	0.01	L-threonine/L-serine transporter
830585	2414	***ansB***	**b2957**	0.54	0.02	periplasmic L-asparaginase II
833677	2423	***malK***	**b4035**	0.52	0.00	fused maltose transport subunit, ATP-binding

824954	2426	***ompF***	**b0929**	0.51	0.01	outer membrane porin 1a (Ia;b;F)
829747	2425	***yfiA***	**b2597**	0.51	0.00	cold shock protein associated with 30S ribosomal
832241	2424	*dppA*	b3544	0.51	0.03	dipeptide transporter
824400	2427	*sucD*	b0729	0.50	0.01	succinyl-CoA synthetase, NAD(P)-binding, alpha
824351	2431	*sucC*	b0728	0.48	0.02	succinyl-CoA synthetase, beta subunit
834098	2430	***treB***	**b4240**	0.48	0.01	fused trehalose(maltose)-specific PTS enzyme:
825844	2429	***ompW***	**b1256**	0.47	0.03	outer membrane protein W
829903	2428	*srlD*	b2705	0.46	0.00	sorbitol-6-phosphate dehydrogenase
829900	2411	*srlB*	b2704	0.45	0.01	glucitol/sorbitol-specific enzyme IIA component
824360	2412	***sucB***	**b0727**	0.45	0.01	dihydrolipoyltranssuccinase
831015	2409	*tdcB*	b3117	0.44	0.00	catabolic threonine dehydratase, PLP-dependent
824363	2410	*sucA*	b0726	0.43	0.01	2-oxoglutarate decarboxylase, thiamin-requiring
833644	2407	*nrfA*	b4070	0.43	0.01	nitrite reductase, formate-dependent,
829891	2408	*srlE*	b2703	0.42	0.01	glucitol/sorbitol-specific enzyme IIB component
829880	2406	*srlA*	b2702	0.39	0.00	glucitol/sorbitol-specific enzyme IIC component
825817	2405	***adhE***	**b1241**	0.27	0.01	fused acetaldehyde-CoA

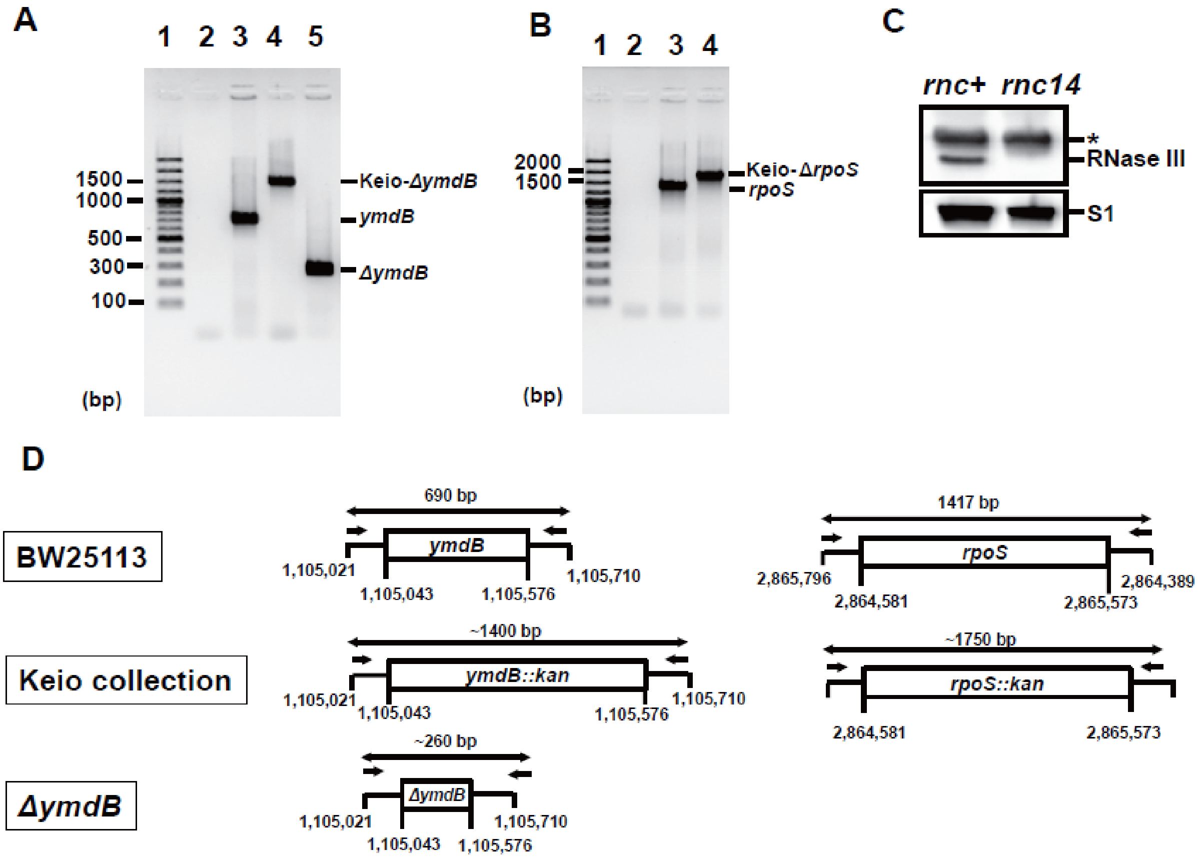

Figure S1. Kim et al.

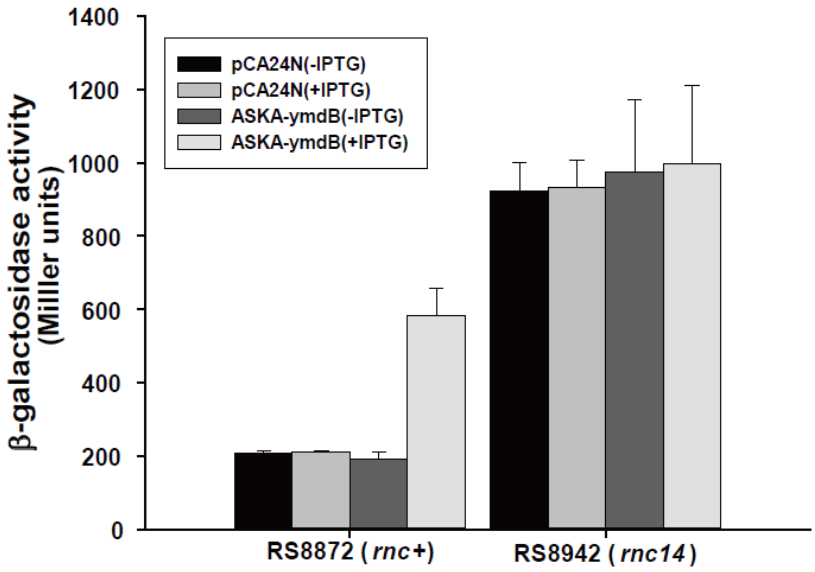

Figure S2. Kim et al.

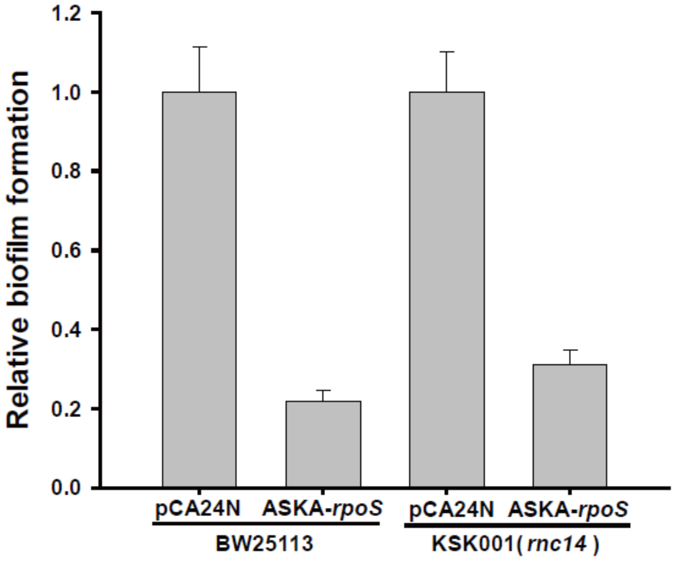

Figure S3. Kim et al.

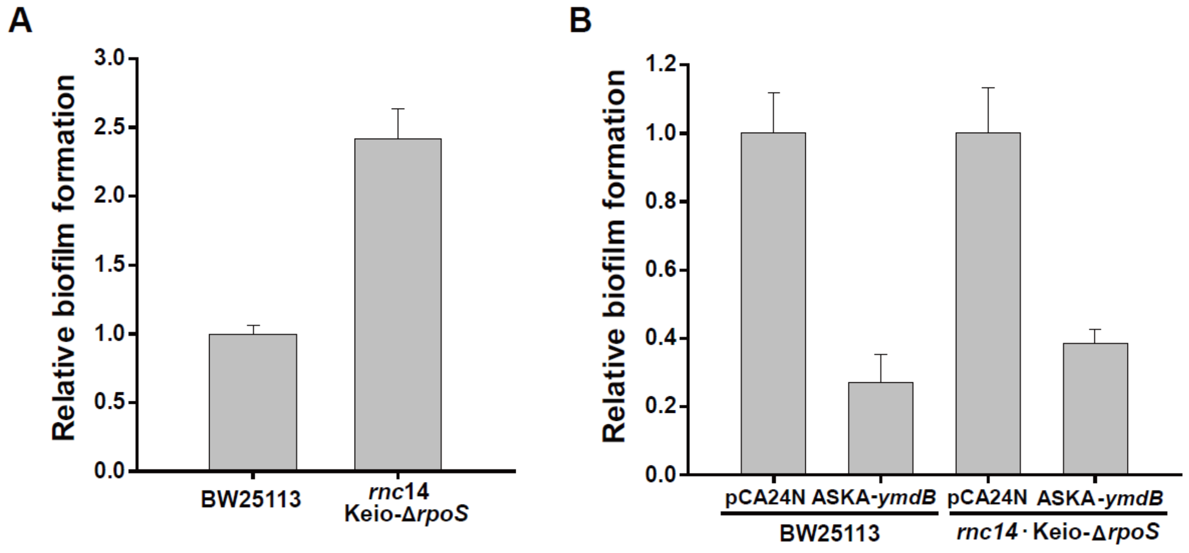

Figure S4. Kim et al.

Figure Legends

Figure S1. Verification of *rpoS*, *ymdB*, and *rnc* mutants. PCR-validation of (A) Keio-*ΔrpoS* or (B) Keio-*ΔymdB* and *ΔymdB*. Colonies of BW25113, Keio-*ΔrpoS, Keio-ΔymdB,* KSK002 (*ΔymdB*), or KSK005 (*rnc14*·Keio-*ΔrpoS*) were amplified using Emerald PCR premix (Takara) and rpoS_F/rpoS_R or ymdB_F/ymdB_R primer sets. Aliquots were resolved in 1.5% agarose gels (Lane 1: 100 bp Plus DNA Ladder (Fermentas), Lane 2: primer pair, lane 3: BW25113, lane 4: Keio-*ΔrpoS* or Keio-*ΔymdB*, lane 5: *ΔymdB* or *rnc14*·Keio-*ΔrpoS*), and photographed using a GelDoc system (E-Graph, ATTO). Bp, base pairs. (C) Schematic representations of PCR regions. The amplified regions of each gene within chromosome MG1655 (GenBank U00096.2) are shown at the bottom of each figure (not to scale). (D) Western-blot analysis verifying RNase III mutation. BW25113 (*rnc+*), KSK001 (*rnc14::Tn10* in BW25113), or KSK005 (*rnc14*·Keio-*rpoS*) cells were grown to OD_{600}=1.0, harvested, and challenged with anti-RNase III or anti-S1. Asterisk (*) indicates non-specific binding by anti-RNase III, as described previously [17].

Figure S2. Dependency of YmdB-mediated down-regulation of RNase III activity upon the presence of RNase III. RNase III activity was assessed according to ß-galactosidase activity resulting from the *pnp'*-*'lacZ* fusion in RS8872 (*rnc+*) or RS8942 (*rnc14::Tn10*) cells containing either pCA24N (-*gfp*) or ASKA-*ymdB* (-). Mean values (n>3) with standard deviations are shown.

Figure S3. Interdependency of RpoS and RNase III for biofilm formation. Effect of biofilm formation by RpoS was measured in both wild type (BW25113: *rnc+*) and KSK001 (BW25113: *rnc14::Tn10*) containing either pCA24N (-*gfp*) or ASKA-*ymdB* (-) were grown in LB complemented with IPTG (0.1 mM final) and chloramphenicol at 37°C for 24 h. The data shown in the figure are the mean values and errors from n=10.

Figure S4. Dependency of YmdB-mediated phenotype upon the absence of RpoS and RNase III. (A) The effect of biofilm formation by double mutation of RpoS and RNase III. Biofilm formation in both wild type (BW25113: *rnc+rpoS+*) and *rnc14·ΔrpoS* double mutant (KSK005: *rnc14·Keio-ΔrpoS*) was measured. (B) Effect of YmdB overexpression in double mutation of *rnc14· ΔrpoS*. BW25113 or KSK005 cells containing either pCA24N (-*gfp*) or ASKA-*ymdB* (-) were grown in LB complemented with IPTG (0.1 mM final) and chloramphenicol at 37°C for 24 h. Biofilm measurements were performed as described in Methods and the mean values and errors from multiple experiments (n=10) are shown.
